# Contribution of AurkA/TPX2 Overexpression to Chromosomal Imbalances and Cancer

**DOI:** 10.3390/cells13161397

**Published:** 2024-08-22

**Authors:** Federica Polverino, Anna Mastrangelo, Giulia Guarguaglini

**Affiliations:** Institute of Molecular Biology and Pathology, National Research Council of Italy, c/o Sapienza University of Rome, Via degli Apuli 4, 00185 Rome, Italy; fede.polverino@gmail.com (F.P.); anna.mastrangelo@uniroma1.it (A.M.)

**Keywords:** mitosis, aneuploidy, chromosome instability, centrosomes, microtubules

## Abstract

The AurkA serine/threonine kinase is a key regulator of cell division controlling mitotic entry, centrosome maturation, and chromosome segregation. The microtubule-associated protein TPX2 controls spindle assembly and is the main AurkA regulator, contributing to AurkA activation, localisation, and stabilisation. Since their identification, AurkA and TPX2 have been described as being overexpressed in cancer, with a significant correlation with highly proliferative and aneuploid tumours. Despite the frequent occurrence of AurkA/TPX2 co-overexpression in cancer, the investigation of their involvement in tumorigenesis and cancer therapy resistance mostly arises from studies focusing only on one at the time. Here, we review the existing literature and discuss the mitotic phenotypes described under conditions of AurkA, TPX2, or AurkA/TPX2 overexpression, to build a picture that may help clarify their oncogenic potential through the induction of chromosome instability. We highlight the relevance of the AurkA/TPX2 complex as an oncogenic unit, based on which we discuss recent strategies under development that aim at disrupting the complex as a promising therapeutic perspective.

## 1. Introduction

Aneuploidy, a term coined by Gunnar Täckholm in 1922, refers to deviations from an exact multiple of the haploid set of chromosomes; it is a hallmark of cancer and is often the result of defective chromosome segregation occurring during mitosis [1,2]. The most common mitotic error that leads to aneuploidy is the establishment of a merotelic attachment, which refers to a kinetochore/microtubule (KT/MT) aberrant interaction where a single KT becomes attached to MTs extending from both spindle poles [3]. This incorrect attachment is not detected by the spindle assembly checkpoint (SAC)—the safety mechanism that monitors accurate chromosome segregation in mitosis—and promotes the formation of lagging chromosomes [1]. Lagging chromosomes, which remain at the cell equator in anaphase, lead to the formation of daughter cells with chromosome gains or losses, and can generate micronuclei in the resulting interphase. Besides aneuploidy, chromosomal instability (CIN) is detected in many cancer types and refers to the rate of karyotypic changes along cell generations; however, while CIN leads to aneuploidy, not all aneuploid cells exhibit CIN, with some maintaining a stable karyotype [4]. In cancer genomes, chromosome aberrations—both numerical and structural—are prominent features [5]. Several studies have deepened our understanding of how structural rearrangements activate oncogenes and deactivate tumour suppressors, influencing tumour development and resistance to therapy [6,7,8]. The role of chromosome numerical variations in tumorigenesis, particularly of specific whole-chromosome aneuploidies, is also actively investigated [9].

Several mitotic factors are deregulated in cancer. Among them, the serine/threonine kinase AurkA and its major regulator, TPX2, have been found to often be co-overexpressed [10] and have been linked to CIN and aneuploidy generation as well as maintenance [11]. Here, we review the existing evidence about the mechanisms through which these two key mitotic regulators—when altered—lead to aneuploidy and CIN, both as single units and as an oncogenic complex. We also summarise innovative approaches to target the AurkA/TPX2 complex that are currently being tested and evaluated as therapeutic strategies.

## 2. Contribution of AurkA Kinase Deregulation to Aneuploidy and CIN

AurkA belongs to the Aurora kinase family, which in humans consists of three members with key regulatory roles in cell division [12,13]. Aurora kinases share a highly conserved catalytic domain at their C-termini, and variable N-terminal regions displaying regulatory domains [13]. During mitosis, the functional diversity between AurkA and AurkB ensures the fidelity of chromosome segregation and is provided by their distinct localisation patterns, which result in the phosphorylation of a distinct set of targets [12]. AurkA is primarily located at the centrosomes and spindle pole MTs, and is involved in multiple processes including centrosome maturation and separation, spindle assembly, chromosome alignment, and segregation [12,13,14,15]. AurkB localises at centromeres, kinetochores, and the spindle midzone, and constitutes the enzymatic component of the chromosomal passenger complex (CPC). It acts in the regulation of KT/MT attachments, SAC function, chromosome segregation, and cytokinesis [12,13,16].

Aurora kinases are frequently overexpressed in cancer [17]. While the oncogenic potential of AurkA has been proposed since its first identification in human cells [18], the link between AurkB and cancer was initially less explored. Here, we will focus on the AurkA family member, and we direct the reader to recent reviews [19,20] for an updated view of AurkB in cancer and as a therapeutic target. The overexpression of AurkA in a wide range of solid and haematological tumours has been described as a consequence of the amplification of the *AURKA* gene or dysregulation of the upstream signalling pathways that control AurkA expression [10,18,21,22]. Some evidence exists positively correlating AurkA overexpression with CIN in tumours and cancer cells [23,24,25]. Based on AurkA relevance in the regulation of the mitotic process, AurkA overexpression was expected to induce aneuploidy through chromosome mis-segregation [26,27]; however, AurkA has multiple mitotic—and newly revealed interphasic—roles that may contribute to generating a complex context [15,28,29]. Thus, how AurkA overexpression leads to aneuploidy and whether this represents a driver event in the tumorigenic process is still under active investigation. We report below existing evidence supporting the link between AurkA overexpression, altered mitotic events related to centrosomes, kinetochore proteins and MT dynamics, and the induction of aneuploidy or chromosome instability.

### 2.1. Effects of AurkA Overexpression on Centrosome Amplification and Fragmentation

The centrosomal localisation of AurkA, the identification of its centrosomal targets [13,14,15], and the frequent occurrence of centrosome abnormalities in cancer cells [30,31] suggest this as a potential route to chromosomal instability following AurkA overexpression. Indeed, the frequent occurrence of supernumerary centrosomes in cancer samples with high AurkA levels was reported [32]. Overexpressing AurkA in nontransformed murine cells, either immortalised cell lines or MEFs from transgenic mice, led to 10–20% of cells having >2 *γ*-tubulin foci [33,34], and higher occurrence was observed after AurkA overexpression in human cancer cell lines [35,36,37]. The lack of centriolar staining in most studies does not allow to unambiguously assess whether these foci always represent supernumerary centrosomes. Interestingly, Meraldi and colleagues [35] performed specific assays to demonstrate that AurkA overexpression does not cause centrosome overduplication, and proposed cytokinesis failure and the resulting polyploidisation as the route to centrosome amplification in the presence of high AurkA levels. Indeed, others reported the concomitant presence of multiple centrosomes and tetraploid cells in AurkA-overexpressing conditions [38,39,40]. In nontransformed human cells, we recently reported that, upon AurkA overexpression, the pericentriolar material (PCM) fragments in mitosis, although centrioles remain in most cases normal in terms of number and organisation [41]. Still, no cytokinesis failure was observed, similarly to what was recorded in human nontransformed MCF10A cells [38]. The variability among cell lines in terms of the extent of supernumerary PCM foci and of polyploidisation suggests that other factors may cooperate in phenotype generation. Interestingly, in some cases the occurrence of supernumerary centrosomes is exacerbated in p53-null cells [35,39]. This may reflect an impairment of the p53-mediated surveillance mechanism, limiting the proliferation of tetraploid cells with supernumerary centrosomes in nontransformed cells [42,43,44], or a more complex scenario, given that the loss of p53 also yields centrosome amplification (>2 *γ*-tubulin spots, each containing a centriole pair) and fragmentation (>2 *γ*-tubulin spots, with no sign of centrosome amplification) [45], while AurkA overexpression weakens p53-dependent responses [41,46]. Finally, the intriguing observation that AurkA inactivation limits the clustering of supernumerary centrosomes [47] leaves the open possibility that the contribution of AurkA overexpression to abnormal centrosome numbers also involves its clustering-promoting ability. Collectively, these observations, although clearly showing a connection between AurkA overexpression and centrosome abnormalities, potentially contributing to chromosome imbalances, do not indicate a unique and direct mechanism for their generation.

### 2.2. Effects of AurkA Overexpression on MT Dynamics and Chromosome Congression

Alongside the well-known AurkA functions in centrosome maturation and spindle pole formation (extensively reviewed elsewhere, such as in [13,14]), additional roles have emerged in MT regulation and function, which may be relevant in the context of chromosomal instability in AurkA-overexpressing tumours. Some of these functions have been linked to a minor fraction of AurkA localised to centromeres/KTs in a manner that depends on the KT serine-threonine kinase Bub1 and the CPC component INCENP [15,48]. The mechanisms underlying these AurkA roles rely on the finely tuned regulation of target phosphorylation levels, normally counteracted by phosphatases. Although not always experimentally demonstrated, it can be envisaged that AurkA overexpression disrupts this fine regulation, thereby promoting incorrect chromosome segregation and resulting in CIN. The best-characterised AurkA targets in these processes are briefly described below.

In 2008, it was reported that AurkA localises to newly formed MTs in several sites near the KTs/chromatin in Hela cells after nocodazole release. AurkA-depleted cells showed decreased MT nucleation at these sites and the failure of MT attachment to the KTs, suggesting a role of AurkA in KT/chromatin-associated MT formation in mammalian cells [48]. Subsequent evidence supported the role of AurkA in the control of MT assembly and dynamics through the phosphorylation of the MT-associated protein HURP and the k-fiber-localised factor MCRS1. Maintaining the temporal or spatial balance between phosphorylated/unphosphorylated forms of these proteins is crucial to ensure correct MT regulation. MCRS1 is unphosphorylated at Ser35/36 in the early stages of spindle assembly, protecting chromosomal MTs from depolymerisation and stabilising them. In metaphase, AurkA-dependent MCRS1 phosphorylation on Ser35/36 is required for its function at the minus ends of the k-fibers, establishing their proper dynamics [49]. Likewise, a balance of phosphorylated HURP at KTs and dephosphorylated HURP at MT minus ends close to the centrosomes is maintained by AurkA-dependent phosphorylation and PP1/PP2A-associated dephosphorylation [50], and is required for the nucleation and stabilisation of KT MTs. Interestingly, a direct link in colon cancer cells has been demonstrated between AurkA overexpression and increased MT assembly rates, which in turn induce CIN. Consistently, elevating AurkA levels and activity in a panel of chromosomally stable cancer cell lines yielded lagging chromosomes and CIN, without a significant increase of supernumerary centrosomes, likely via the pathway regulated by the interacting chTOG MT polymerase and TACC3 coiled-coil protein [24]. Consistent with the role of AurkA in regulating MT dynamics, the stable and inducible overexpression of AurkA in human immortalised nontransformed hTERT RPE-1 cells yielded spindle misorientation, associated with an increased length (but not overall abundance) of astral MTs, which could be corrected by treating cells with subnanomolar doses of Taxol, known to rescue MT dynamic defects [51]. Still, no lagging chromosomes or micronuclei were present, and the mild aneuploidy observed was likely due to the presence of anaphase chromosome bridges [41]; these observations suggest important differences in the effects deriving from elevating AurkA levels in transformed and nontransformed cells.

The function of AurkA in regulating KT/MT attachments and chromosome congression also relies on the phosphorylation of centromere and KT components. The first described AurkA phosphorylation target at centromeres was CENP-A [52], a histone variant defining the centromeric regions on chromosomes. In Hela cells, AurkA phosphorylates CENP-A on a conserved serine residue (Ser7) within its N-terminal tail [52,53]. CENP-A-Ser7 phosphorylation by AurkA in prophase is required for proper mitotic progression, and the expression of a CENP-A non-phosphorylatable mutant leads to aberrant chromosome segregation and decreased cell viability. AurkA-dependent CENP-A phosphorylation has been proposed to stabilise the chromatid cohesion regulator shugoshin protein 1 (Sgo1) at centromeres, offering protection against chromosome fatigue until all chromosomes are bioriented [53]. Within the process of chromosome biorientation and congression, the correction of maloriented KT/MT attachments in the vicinity of spindle poles was proposed to be partly dependent on an AurkA activity gradient [54], which may be altered in AurkA-overexpressing conditions. Interestingly, a recent work indicates the KT-associated Mps1 kinase as an important regulator of this gradient, via the proximity-dependent potentiation of AurkA auto-phosphorylation; this constitutes an intriguing link to be explored in light of the observation that Mps1 overexpression favours the proliferation of aneuploid cancer cells [55,56]. AurkA directly phosphorylates the kinesin motor protein CENP-E at Thr422 in the proximity of spindle poles and promotes CENP-E activity counteracting its binding to the phosphatase PP1, which dephosphorylates CENP-E after chromosome congression to ensure stable attachment and biorientation [57,58]. In addition, it has been demonstrated that AurkA plays a role in correcting erroneous KT/MT attachments by phosphorylating the KT-associated protein Hec1/Ndc80, resulting in a reduced affinity of the Ndc80 complex for MTs and the destabilisation of KT/MT attachments [54,59,60,61]. The phosphorylation by AurkA of Hec1/Ndc80 on Ser55 and Ser69 is crucial for chromosome oscillation. The attenuation of this phenomenon, as observed in cancer cells, leads to CIN. Consistently, cells with inhibited or depleted AurkA exhibit decreased Hec1/Ndc80 phosphorylation during prometa- and metaphase, reduced chromosome oscillation, and an increased incidence of lagging chromosomes [59,60]. It has been proposed that AurkA/TPX2 phosphorylates Hec1/Ndc80 in early mitosis to promote the rapid turnover of KT/MT attachments, while AurkA/INCENP maintains this phosphorylation when chromosomes are aligned at the metaphase plate and KTs are stably attached to MTs, facilitating the dynamics of KT/MTs and preventing chromosome segregation errors [59]. Interestingly, Sobajima et al. [61] linked amplified AurkA activity in PPP6C phosphatase KO cells with increased Hec1/Ndc80 phosphorylation, spindle defects, and micronucleation, suggesting that Hec1/Ndc80 is a critical target of AurkA/TPX2 during spindle formation and chromosome segregation (see the following sections).

Finally, besides influencing the correct biorientation and congression of mitotic chromosomes, high levels of AurkA can also interfere with the response to erroneous KT/MT attachments. Early studies indicated that AurkA overexpression yields the overriding of the SAC in U2OS cancer cells treated with nocodazole [62], an effect that can become therapeutically relevant when antimitotic drugs are used. Indeed, parallel work showed that AurkA overexpression interferes with the SAC and confers resistance to apoptosis induced by Taxol, a chemotherapeutic agent that prevents MT depolymerisation [40].

**Figure 1:**
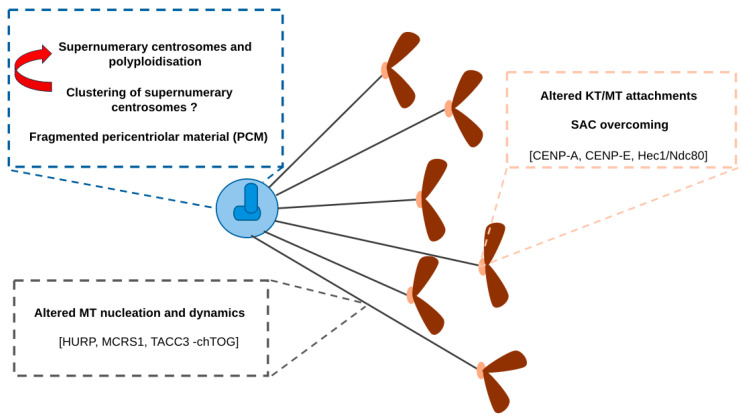
Mechanisms through which AurkA overexpression can yield chromosome segregation defects and aneuploidy by acting at the level of centrosomes (top-left box), MTs (bottom-left box), and centromeres/KTs (right box) are summarised. Described targets are also indicated.

Together, the results summarised so far and schematised in Figure 1 indicate that AurkA overexpression may promote CIN through multiple routes via mitotic defects. Still, results in nontransformed cells and the variability recorded among different cell lines suggest that the cellular background cooperates and that AurkA overexpression alone may not be sufficient to establish a chromosomally unstable context.

## 3. TPX2 and CIN: A Tangled Link

TPX2 is a MT-associated protein, with its expression and protein levels finely regulated during the cell cycle, peaking in G2 and mitosis, and sharply decreasing at mitotic exit via APC/C- and proteasome-dependent degradation [63,64]. TPX2 localises in the nucleus before nuclear envelope breakdown, and at the spindle MTs in mitosis. It was identified as an essential spindle assembly regulator in Xenopus egg extracts and human cells [65,66,67]. It was the first identified SAF (spindle assembly factor) downstream of the Ran network in mitosis [66,68]. The mechanisms through which TPX2 exerts its essential mitotic roles have been clarified through the years and mostly rely on its ability to promote MT nucleation and branching [69,70,71] and to interact with kinesins such as Eg5 and Kif15 [72,73]. A key contribution to TPX2 functions derives from it being the best-characterised AurkA regulator [68,74,75,76]. The N-terminal region of TPX2 is required for binding to the catalytic domain of AurkA; the interaction induces a conformational change in the kinase, promoting its activation [75,77]. Furthermore, TPX2 mediates AurkA localisation to spindle MTs [74,78] and is required for AurkA protein stabilisation in the early phases of mitosis [76].

TPX2 overexpression has been associated with cancer since its discovery [79], is reported in several cancer-specific signatures (see, for example, [80,81,82]), and almost two decades ago it was found to display the highest correlation score in a gene signature of association with chromosomally unstable tumours [83]. Carter and colleagues obtained a CIN score for more than 10,000 genes based on gene expression data in multiple tumour datasets previously characterised for overall chromosomal imbalances [83]. The 70 top-ranking genes (CIN70 signature) in the list were mostly involved in faithful DNA replication and chromosome segregation, and *TPX2* is the gene with the highest CIN score. The observation that many identified genes, among which is *TPX2* itself, correlate with cell proliferation and the cell cycle raised the possibility that their high expression in CIN tumours was a consequence of high proliferation; importantly, authors were able to demonstrate that the prognostic ability of the CIN70 signature was not solely reflecting the proliferative feature of cancer cells.

A recent study on breast cancer samples, while confirming the association of high levels of TPX2 with chromosomal imbalances, challenges the value of TPX2 as an independent predictor of CIN [84]. First, the correlation between TPX2 gene expression data and protein levels was confirmed in a cohort of primary breast cancer samples. Then, tumours with high nuclear TPX2, revealed by immunohistochemical analysis, were analysed for aneuploidy features, assessed by FISH. High levels of nuclear TPX2 positively correlated with increased ploidy and CIN compared with low nuclear TPX2 samples. Interestingly, a positive correlation with *TP53* mutation was also observed; however, linear regression models, including multiple variables, led the authors to conclude that TPX2 had no significant independent association with CIN; the correlation between high nuclear TPX2 and the expression of the proliferation marker Ki67 rather suggested that TPX2 directly correlates with an increased proliferation status.

**Figure 2:**
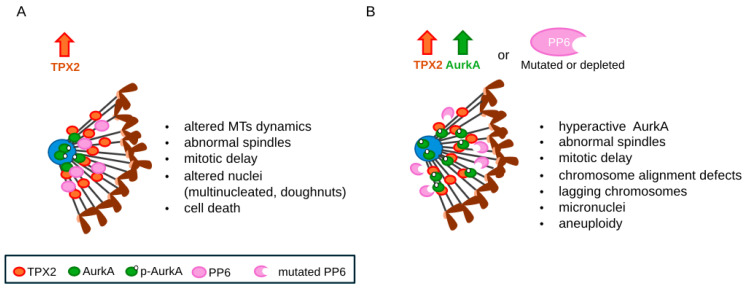
Effects of overexpressing TPX2 alone (**A**) or the whole AurkA/TPX2 complex (**B**). PP6 mutations harboured by cancer cells or PP6 depletion yield a cellular effect comparable to AurkA/TPX2 overexpression, by impairing the dephosphorylation of AurkA Thr288 within the AurkA/TPX2 complex (**B**). The distribution of TPX2, AurkA, and auto-phosphorylated AurkA (p-Aurka, Thr288) on the mitotic spindle is schematised on the left in each panel (a half spindle is sketched; the centrosome is in light blue, chromosomes in brown, the black lines represent MTs).

Studies performed in cancer cell lines show that TPX2 overexpression yield mitotic arrest, spindle defects, aberrant daughter cells, and cell death [67,85,86], highlighting the importance of balanced TPX2 expression for cell division and proliferation, while not directly relating high TPX2 levels to chromosomal instability (Figure 2A). Interestingly, no mouse models for TPX2 overexpression are reported, while TPX2-haploinsufficient mice display chromosomal imbalances and a shorter tumour-free lifespan, with increased probability to develop spontaneous tumours [87]. Thus, non-conclusive results on the role of TPX2 overexpression per se in the generation of CIN or aneuploidy exist, suggesting complex underlying mechanisms that need in-depth investigation.

To contribute to the understanding of the role of TPX2 overexpression in chromosome instability, we raised TPX2 levels in human nontransformed hTERT-RPE-1 cells. Consistent with its relevance in spindle MT nucleation and organisation, and with results previously obtained in cancer cells, TPX2 overexpression yields MT organisation defects, associated with prolonged mitosis, and hyperstable MTs that are more resistant to nocodazole treatment or ice-induced depolymerisation compared with control cells [51,88]. Unexpectedly, we did not detect a significant occurrence of chromosome segregation errors in ana- and telophase or micronuclei in the subsequent interphases (Figure 2A). The most represented defect in cells deriving from TPX2-overexpressing mitoses were doughnut-shaped nuclei with an unsealed nuclear envelope and a peculiar distribution of chromosomes and cellular organelles [88]. This phenotype may represent a non-canonical route to genome rearrangements and aneuploidy in a nontransformed background, as has also been independently proposed [89,90], although this possibility requires further investigation.

Collectively, these results suggest that the involvement of TPX2 overexpression in aneuploidy or CIN induction could also rely on alternative mechanisms to canonical chromosome mis-segregation events, or on the co-deregulation of other factors.

Supporting the latter possibility, Rohrberg and colleagues [91] found that Myc, whose overexpression in nontransformed hTERT RPE-1 cells induces spindle MT defects and aneuploidy, can transcriptionally upregulate TPX2, abolishing its cell cycle regulation. Interestingly, in this condition, hTERT RPE-MYC cells depend on high TPX2 levels for survival, since TPX2 silencing leads to cell death in mitosis. In line with this result, high-Myc xenograft tumours are not able to grow after the knockdown of TPX2 expression by TPX2-shRNA [91]; therefore, this study supports the possibility that the involvement of TPX2 overexpression in aneuploidy induction may reflect its functional or physical interaction with other factors. In the following paragraph, we will pursue this possibility further, by summarising evidence on the contribution of AurkA/TPX2 co-overexpression to chromosome instability.

## 4. AurkA/TPX2 Complex Deregulation Promotes Genome Instability in Cancer

More than ten years ago, we highlighted the frequent co-overexpression of AurkA and TPX2 in cancer, and proposed that it holds oncogenic potential, resulting in the formation of an oncogenic holoenzyme [10]. This initial hypothesis has been supported by experimental evidence throughout the following years [92,93], although a direct and unequivocal demonstration is still missing. Here, we recapitulate the findings that link the deregulation of the AurkA/TPX2 complex with aneuploidy, CIN, cell transformation, and drug resistance, thus supporting its pro-tumorigenic role.

Starting from the CIN70 signature (see above), Szas and colleagues interrogated 10 publicly available breast cancer datasets and defined a smaller signature of genes with equivalent information in terms of CIN correlation, which was termed CIN4 [11]. Interestingly, both *AURKA* and *TPX2* are included in this minimal signature, which directly correlates with an increased DNA index (aneuploidy vs. euploidy) in samples from breast cancer patients, and is able to stratify grade 2 tumours for a good or bad prognosis.

An independent study [86] identified *TPX2* among the top 250 genes related to genomic instability, through transcriptome-wide association analysis. They observed that, in BRCA2-deficient breast cancer cells, the silencing of either AurkA or TPX2 causes a reduction in cell viability compared to BRCA2-proficient cells, suggesting that the survival of genomically unstable cancer cells depends on the AurkA/TPX2 signalling axis.

An additional link between the deregulation of the AurkA/TPX2 complex and chromosomal instability in tumours comes from studies on the phosphatase PP6, which has been demonstrated to be the AurkA T-loop phosphatase when the kinase is complexed with TPX2 [94]. PP6 depletion therefore leads to hyperactive TPX2-bound AurkA and may mimic the condition of AurkA/TPX2 complex overexpression in cancer (Figure 2B). Interestingly, PP6 is frequently mutated in melanoma, leading to the loss of its catalytic function or destabilisation [95]. Consistently, tumour cells carrying PP6 mutations and lacking PP6 catalytic activity display increased activity (by 200%) of AurkA in the complex with TPX2. PP6-depleted HeLa cells undergo prolonged mitosis associated with chromosome alignment defects that culminate in chromosome segregation errors and micronuclei; micronucleation and aneuploidy, detected by FISH, are also observed in PP6-mutated melanoma cells ([61,95]; Figure 2B). Under both conditions, treatment with the AurkA kinase inhibitor Alisertib mitigates the micronucleation phenotype; similar results are also achieved with TPX2 RNAi in PP6-depleted HeLa cells [94]. Together, these results strengthen the notion that the regulation of the complex is essential for chromosome segregation fidelity and thus to avoid CIN.

To directly address the relevance of deregulating the whole AurkA/TPX2 complex with respect to aneuploidy induction, we have recently overexpressed in a nontransformed background either AurkA alone or AurkA/TPX2 [41]. We first revealed that AurkA overexpression per se is not sufficient to hyperactivate AurkA and thus induce strong aneuploidy in hTERT RPE-1 cells. Instead, the overexpression of the whole complex induces AurkA hyperactivity, leading to chromosome segregation errors, such as lagging chromosomes, micronuclei, and aneuploidy (Figure 2B). Consistent with the results discussed above, the micronuclei generated in hTERT RPE-1 cells upon AurkA/TPX2 overexpression are rescued by limiting AurkA hyperactivation with low doses of Alisertib that restore p-Thr288-AurkA at “near physiological” levels. Importantly, resistance to cell cycle arrest induced by nocodazole treatment/release is also observed, supporting the hypothesis that AurkA/TPX2 overexpression favours the generation and propagation of CIN cells [41].

Interesting evidence is also emerging about the contribution of AurkA/TPX2 complex overexpression to drug resistance. Shah and colleagues revealed that AurkA hyperactivation due to TPX2 overexpression is responsible for the acquired resistance to the EGFR tyrosine kinase inhibitor in non-small-cell lung cancer cell lines and patient-derived cells [96]. HeLa and MDA-MB-231 cells overexpressing AurkA also display a limited response to taxanes [40,97], supporting a role of AurkA overexpression in chemoresistance. On the other hand, the observation that high levels of AurkA and TPX2 are associated with taxane-based radiosensitisation [98], a strategy currently used in clinical protocols for a large spectrum of tumours, suggests that their involvement in therapeutic responses may depend on tumour or treatment specificity, highlighting the need for further investigation.

The known interplay between AurkA and p53 (extensively discussed in [46]) may also be relevant for the propagation of chromosomal unstable cells and resistance induction. Evidence of the role of TPX2 in regulating this interplay has been described only in Xenopus egg extracts, showing that p53 can inhibit AurkA activity only in the absence of TPX2 [99], and that TPX2 contributes to AurkA-dependent p53 phosphorylation [100]. Furthermore, TPX2 has been shown as an interactor of both 53BP1 [101] and PARP1 [102]. Studying the oncogenic potential of AurkA/TPX2 complex overexpression in a nontransformed cellular background (see above), we recently observed that AurkA/TPX2 co-overexpression not only induces aneuploidy but also impairs p53 accumulation and function upon different stimuli. Interestingly, we also reported that AurkA/TPX2-co-overexpressing cells can overcome the expected cell cycle arrest upon independent mitotic stresses, such as prolonged mitosis and micronuclei induction, by keeping low levels of both p53 and its downstream target, p21 [41]. These results support the notion that AurkA acts as an oncogene and that TPX2 is essential for this function through several routes.

## 5. The AurkA/TPX2 Complex as a Therapeutic Target

As described above, the AurkA/TPX2 complex is overexpressed in several tumour types (breast, lung, prostate, ovarian, and others) and contributes to aneuploidy as well as CIN, favouring tumour heterogeneity and drug resistance. On these bases, the AurkA/TPX2 complex is emerging as a potential therapeutic target in cancer. Here, we summarise the innovative approaches developed so far aimed at inhibiting the binding of TPX2 to AurkA within the wider framework of AurkA inhibitors.

AurkA inhibitors directed at the ATP binding site have been largely evaluated in pre-clinical studies and clinical trials, and extensively reviewed elsewhere [17,103]. So far, their use in clinical studies has proved less effective than expected, highlighting the need for new therapeutic approaches. One explored strategy is a combination treatment with inhibitors of different key cell cycle regulators, which has provided some promising results in cellular studies, depending on the tumour type and experimental protocol [17,104,105,106,107]. Furthermore, exploiting AurkA synthetic lethal interactions with oncosuppressors frequently mutated in cancer may be a route with which to improve AurkA-based therapeutic strategies [108].

In recent years, interest has also grown in the direction of non-mitotic and kinase-independent AurkA roles that are now emerging as a route for both tumour onset and progression, and may partly account for the poor efficacy of AurkA inhibitors in clinical trials [28,29]. Targeting AurkA by using PROTACs (proteolysis-targeting chimaeras), bifunctional small molecules that promote the proximity of the target to specific ubiquitin E3 ligases, thus leading to its ubiquitination and proteasome-dependent degradation [109], may confer advantages over the use of AurkA catalytic inhibitors. Indeed, PROTACs would tackle both catalytic and non-catalytic AurkA functions; furthermore, while kinase inhibitors have an intrinsic level of promiscuity due to the high conservation of the ATP binding site, PROTACs can be designed with high specificity for the target. Studies carried out using AurkA-directed PROTACs based on the AurkA inhibitor Alisertib have pointed out that cellular effects are distinct from those induced by AurkA catalytic inhibitors and suggested the possibility of targeting specific AurkA subcellular pools. In cancer cells treated with JB170, S-phase arrest/delay was observed, differently from the G2/M enrichment observed upon Alisertib treatment [110]. Interestingly, targeting AurkA interphasic and mitotic pools, using specific PROTACs directing the kinase to ubiquitin ligases with cell cycle-regulated expression, has been proposed as a feasible strategy [111]. An independent study reported the elimination of the mitotic spindle-associated fraction of AurkA, interacting with TPX2, in U2OS and hTERT RPE-1 cells treated with PROTAC-D, while preserving the centrosomal fraction [112]. The possibility to target the AurkA/TPX2 pool is particularly interesting in light of our recent observation that the interaction between AurkA and TPX2 is also essential in interphase to stabilise and protect AurkA from degradation, thus leading to AurkA nuclear accumulation [113]. Therefore, inhibiting the formation of the complex could be relevant for AurkA non-mitotic and nuclear oncogenic functions [88]. Efforts to develop allosteric inhibitors of AurkA, preventing its binding to TPX2, are discussed below.

**Figure 3:**
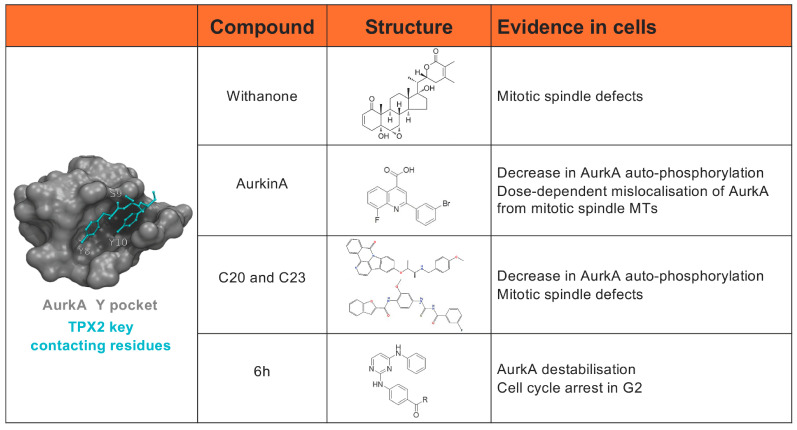
PPI inhibitors described to target the AurkA/TPX2 complex and tested in cultured cells are listed, together with their structures and observed effects. The AurkA hydrophobic pocket binding residues 8–10 of TPX2, as well as the AurkinA, C20/23, and 6h compounds, is shown on the left [114,117,118,119].

The solved structure of the catalytic domain of AurkA in complex with the first 43 aa of TPX2 [75] provided relevant information on the interaction interface, which guided the design of strategies with which to identify AurkA/TPX2 protein-protein interaction (PPI) inhibitors (listed in Figure 3). The first described PPI inhibitor was Withanone, a natural compound that blocks intermolecular hydrophobic interactions between TPX2 and AurkA involved in the formation of the complex, hindering its functionality and impairing mitotic spindle organisation in MCF7 cancer cells [114]. Subsequently, different groups directed their attention to the hydrophobic pocket of AurkA, which contacts aa 8–10 of TPX2 (Figure 3, left). A synthetic vNAR single-domain antibody, v-NAR-D01, was shown to bind and inhibit AurkA. The crystal structure of v-NAR-D01/AurkA reveals an allosteric mode of action antagonistic to the mechanism by which TPX2 activates the kinase; indeed, v-NAR-D01 interferes with TPX2 binding to AurkA by interacting with the same region on the kinase. Although vNAR-D01 lacks the necessary binding strength to be effective in cell-based assays, it provided a rational basis for designing allosteric AurkA inhibitors by structure-guided approaches [115]. The druggability of the AurkA/TPX2 interaction interface was supported by a X-ray crystallography-based fragment screen, and the hydrophobic pocket, named the Y-pocket, of AurkA, normally occupied by the Tyr8-Ser9-Tyr10 motif of TPX2, emerged as a promising target site [116].

Small molecules directed against this pocket have been described. First, AurkinA was identified by high-throughput screening using a library of 17,000 rationally selected compounds, and was shown to interfere with AurkA/TPX2 complex formation [117]. AurkinA binding induces conformational changes in the kinase that inhibit its catalytic activity, both in vitro and in cells, without preventing the binding of ATP to the active site. In cells, AurkinA causes the dose-dependent mislocalisation of AurkA from mitotic spindle MTs, supporting its action as an AurkA/TPX2 PPI inhibitor. In a parallel study, a virtual screening approach was used and two drug-like small molecules capable of interfering with the formation of the AurkA/TPX2 complex in vitro and in cells were identified. U2OS osteosarcoma cells treated with those compounds displayed a decrease in AurkA auto-phosphorylation and mitotic spindle defects consistent with AurkA activity inhibition [118]. Starting from these molecules, a novel allosteric AurkA inhibitor (6h) with a N-benzylbenzamide backbone was recently designed [119], with the ability to inhibit AurkA/TPX2 binding in vitro. In cells, 6h treatment led to AurkA destabilisation, compatible with a lack of TPX2 binding, and affected AurkA non-catalytic functions. Interestingly, a differential G2 arrest was observed in colon cancer (HT29 and HTC116) —with respect to other cancer— cells.

## 6. Conclusions

The results of the reported studies overall sustain the idea that the condition of abnormally high levels of the AurkA/TPX2 complex leads to the hyperactivation of AurkA and exacerbates the defects induced by the overexpression of the single components, thus favouring chromosome segregation errors, aneuploidy, and CIN propagation that are functional features for both cell transformation and resistance occurrence. Furthermore, they support the feasibility of targeting the AurkA/TPX2 complex and they pave the way for further research on the development of effective anticancer therapies specifically affecting oncogenic AurkA/TPX2 functions and thus mitigating mitotic defects, aneuploidy, and chromosomal instability in cancer cells.
